# EMERGE: Early antiMicrobial stEwaRdship for GEneral medicine patients—targeting inpatient intravenous therapy greater than 24 hours

**DOI:** 10.1017/ash.2026.10313

**Published:** 2026-03-27

**Authors:** Sharmila Khumra, Ashmitha Thomas, Sara Vogrin, Adele Batrouney, Kate Lowe, Shayne Camilleri, Nicholas Jones, Jason A. Trubiano, Satwik Motaganahalli, Gemma Reynolds

**Affiliations:** 1 Department of Infectious Diseases and Immunology, Austin Health, Melbourne, Victoria, Australia; 2 Department of Pharmacy, Austin Health, Melbourne, Victoria, Australia; 3 Department of Infectious Diseases, Royal Darwin Hospital, Darwin, Northern Territory, Australia; 4 Department of General Medicine, Austin Health, Melbourne, Victoria, Australia; 5 Department of Infectious Diseases, Doherty Institute, University of Melbourne, Melbourne, Victoria, Australia; 6 National Centre for Infections in Cancer, Peter MacCallum Cancer Centre, Melbourne, Victoria, Australia

## Abstract

**Objective:**

Early review of intravenous (IV) antimicrobial therapy is central to antimicrobial stewardship (AMS), however scalable models for general medical patients are limited. We evaluated a pharmacist-led digital intervention to optimize IV antimicrobial prescribing.

**Methods:**

A prospective, quasi-experimental before-and-after study was conducted between May 2022 to February 2023 across six general medicine units at a tertiary hospital. AMS recommendations were delivered electronically via Microsoft Teams®. Adult inpatients receiving IV antimicrobials for >24 hours were included, excluding those with COVID-19, under Infectious Diseases consultation or receiving palliative care. The primary outcome was median IV antimicrobial duration. Secondary outcomes included AMS recommendation type, recommendation acceptance, length of stay (LOS), 30-day infection-related readmission, IV therapy recommencement, and inpatient mortality. Antibacterial consumption was analyzed from July 2021 to through December 2024 to evaluate sustained impact.

**Results:**

Among 723 antimicrobial orders (474 treatment episodes in 458 patients), median IV duration was comparable between phases (intensive: 2.75 days; baseline: 3.00 days). LOS was shorter during the intensive phase compared to baseline (5.5 vs 7.6 days; *P* = .04), particularly in patients without bacteremia. Readmissions and mortality were unchanged. Of 400 AMS recommendations, 67% were IV-to-oral switches; overall acceptance was 78%. Piperacillin-tazobactam use declined, and sustained reductions in aminoglycosides, ampicillin and IV flucloxacillin were observed. A reduction in total antibiotic prescribing (combined IV and oral prescribing) was also observed.

**Conclusions:**

The digital pharmacist-led AMS intervention did not reduce IV duration, likely reflecting strong baseline prescribing, but was associated with shorter LOS and a reduction in total antibacterial use. This program offered a scalable, sustainable alternative to resource-intensive face-to-face models.

## Introduction

Improving antimicrobial prescribing in hospitals is a key aim of antimicrobial stewardship (AMS), helping to prevent the emergence of resistant organisms, improve patient outcomes, reduce adverse effects, and promote cost-effectiveness.^1^ Early review of IV antimicrobial therapy (within 24 to 72 hours) offers a critical opportunity to act on new diagnostics,^2,3^ enabling cessation of unnecessary therapy, narrowing spectrum, switching to oral agents, or facilitating hospital-in-the-home care, all of which reduce harm, antimicrobial resistance, and length of stay.^1,2^

Despite these benefits, early IV review is not consistently implemented in practice.^3^ Prescriber checklists,^4,5^ questionnaires,^6–8^ and face-to-face prompts,^4^ have achieved only modest improvements, while infectious diseases (ID) physician-led audit and feedback, are resource-intensive and difficult to scale.^9^ Novel and sustainable models are therefore needed. Pharmacist-led, digital, and hybrid approaches that integrate multidisciplinary expertise with efficient communication offer scalable alternatives.^10,11^ This study evaluates a pharmacist-led digital intervention targeting IV antimicrobial prescribing.

## Methods

We conducted a prospective, quasi-experimental before-and-after study at a tertiary hospital with a well-established AMS program, including postprescription review and feedback rounds,^12,13^ formulary restrictions requiring preauthorization for targeted antimicrobials, local antimicrobial prescribing guidelines,^14^ antifungal stewardship^15^ and an antibiotic allergy delabeling program.^16^ A pharmacist-led, multidisciplinary AMS intervention was implemented across six general medicine inpatient teams, accounting for approximately 100 patients daily. As our hospital does not have an acute inpatient geriatric service, general medicine admissions typically reflect a more elderly, medically comorbid cohort.

The study comprised a 3-month baseline observational phase (May 2022 to August 2022), followed by a 6-month intervention with an intensive phase (August–November 2022, daily weekday reviews) and a maintenance phase (November 2022–February 2023, reviews three times per week). The intensive daily phase was informed by a daily AMS model implemented within our intensive care unit.^17^ The maintenance phase evolved from feedback from general medicine stakeholders indicating that less frequent reviews would be acceptable. Although this phase continued beyond the formal study period until December 2024, antimicrobial use after February 2023 was monitored only at the unit level.

Baseline prescribing was assessed retrospectively by an AMS pharmacist, with questions related to data collection referred to an ID physician for final determination. During the intervention period, inpatients on >24 hours of IV antimicrobials underwent morning chart review. All general medicine inpatients outside of intensive care were included. Following chart review, patients were excluded from AMS recommendations if they were managed by overlapping services: patients treated for COVID-19 (due to a separate AMS program), receiving palliative care, under ID consultation, or prescribed antimicrobials for prophylaxis due to immunosuppression. Findings were synthesized by the AMS pharmacist and ID physician, with recommendations provided electronically via Microsoft Teams® by the pharmacist by midday. The ID physician remained available for subsequent clarifications, further advice, and additional clinical questions between electronic reviews.

The primary outcome was the duration of IV antimicrobial therapy during the intervention phases, compared to baseline. Secondary outcomes included the type and frequency of AMS recommendations,^17^ acceptance of recommendations within 24 hours, antimicrobial use (measured as Days of Therapy [DOT] per 100 Occupied Bed Days [OBD] per month). Additional safety outcomes included hospital LOS, recommencement of IV therapy, infection-related 30-day readmission, and all-cause mortality. Data for the baseline phase were collected retrospectively, while data for the intensive phase were collected prospectively. During the maintenance phase, prospective data collection continued for patients reviewed by the AMS service. For patients receiving antimicrobials who were not formally reviewed during this phase, data were collected retrospectively.

Descriptive statistics are presented as median (interquartile range, IQR) or frequency (percentage). Characteristics across the 3 phases were compared, with the intensive and maintenance phases each compared against the baseline phase, using rank sum test or Fisher’s Exact test. IV duration of each treatment course was compared among phases using linear regression with standard errors adjusted for clustering of treatment courses within patients. Outcome was transformed using natural logarithm to enable better model fit. Results are expressed as exponentiated coefficients (fold difference in antibiotic duration) with 95% confidence intervals (CI). Similar analysis was performed for LOS, while logistic regression was used for binary outcomes. All models were adjusted for sex, urinary tract infection, skin and soft tissue infection and blood culture result. Antibacterial consumption was analyzed using interrupted time series analysis with ordinary least-squared regression and Newey West standard errors. Analysis was performed using Stata 18 (StataCorp LLC, Tx, USA).

## Results

We analyzed 723 antimicrobial orders across 474 treatment courses in 458 patients. Of these, 103 patients comprised the baseline group, 178 were in the intensive phase, and 177 in the maintenance phase, before the switch to unit-level antibacterial monitoring. Median age was 82 years (IQR 72, 88), 45% (n = 207) were female and median Charlson Comorbidity Index was 5 (IQR 4, 7). The most common indications for IV therapy were respiratory tract infections (n = 216, 46%), urinary tract infections (n = 111, 24%) and skin and soft tissue infections (n = 47, 10%) (Table 1).

**Table 1.** Demographics and infections data

	Baseline	Intensive phase	Maintenance phase
Demographics			
Number of patients	103	178	177
Median age (years, interquartile range [IQR])	83 (70, 89)	81 (71, 87)	81 (73, 87)
Gender (female, %)	58 (56%)	78 (44%)	71 (40%)
Median Charlson Comorbidity Index (IQR)	6 (4,7)	5 (4,7)	5 (4,8)
Bacteremia (n, %)	24 (23%)	18 (10%)	20 (11%)
Sites of infection			
Total number of infections	119	192	154
Respiratory tract (n, %)	47 (39%)	93 (48%)	76 (49%)
Urinary tract (n, %)	39 (33%)	38 (20%)	34 (22%)
Skin and soft tissue (n, %)	10 (8%)	28 (15%)	9 (6%)
Intra-abdominal (n, %)	6 (5%)	12 (6%)	9 (6%)
Sepsis of unknown source (n, %)	5 (4%)	6 (3%)	8 (5%)
Fever of unknown source (n, %)	2 (2%)	3 (2%)	7 (5%)
Gram-negative bacteremia, unknown source (n, %)	3 (3%)	4 (2%)	3 (2%)
Gram-positive bacteremia, unknown source (n, %)	1 (1%)	2 (1%)	2 (1%)
CNS (n, %)	2 (2%)	3 (2%)	1 (1%)
Other (n, %)	4 (3%)	2 (1%)	4 (3%)

Median IV antimicrobial duration per treatment course in the baseline phase was 3 days (IQR 2, 4), with no significant change during the intensive (median 2.75 days; IQR 2, 3.66; exp coef 0.96; 95%CI 0.9–1.04; *P* = .332) and maintenance phase (median 3 days; IQR 2, 4; exp coef 1.03; 95%CI 0.95–1.11; *P* = .479), even after adjustment for covariates (gender, antimicrobial indication, and presence of bacteremia, Table 2).

**Table 2.** Primary and secondary outcomes

	Baseline	Intensive Phase	Maintenance phase	*P*-value (intensive vs baseline)*	*P*-value (maintenance vs baseline)*
Primary outcome					
	N = 111 courses	N = 184 courses	N = 179 courses		
Median duration of IV antimicrobial therapy (days, (IQR))	3 (2, 4)	2.75 (2, 3.66)	3 (2, 4)	0.332	0.479
Secondary outcomes					
	N = 103 patients	N = 178 patients	N = 177 patients		
Inpatient mortality, (n, %)	11 (10.7%)	17 (9.6%)	14 (7.9%)	0.574	0.302
Length of stay (median (IQR))	7.6 (4.1, 14.0) (N = 92)^a^	5.5 (3.6,10.1) (N = 161)^a^	7.0 (4.2, 11.4) N = 163)^a^	0.004	0.366
Readmission due to infection within 30 days, (n, %)	4 (4%) (N = 92)^a^	10 (6%) (N = 161)^a^	13 (8%) N = 163)^a^	0.591	0.469
Recommencement of IV antimicrobials within 48 hours of oral switch (n, %)	7 (7%) (N = 95)^b^	2 (1%) (N = 141)^b^	5 (4%) (N = 135)^b^	0.036	0.227

* Statistical significance was set at *P* < 0.05; comparison between groups performed using rank sum test and Fisher’s Exact.

a Denominators reflect the number of patients included in the analysis who survived.

b Denominators reflect the number of patients included in the analysis whose IV antimicrobial therapy was switched to oral.

There were 400 AMS recommendations, most commonly IV-to-oral switch (n = 268, 67%) (Figure S1). 78% (n = 311) of recommendations provided were accepted within 24 hours. Ceftriaxone (n = 138, 35%), benzylpenicillin (n = 60, 15%), amoxicillin/clavulanate (n = 48, 12%), flucloxacillin (n = 42, 11%) and ampicillin (n = 31, 8%), were the most frequent agents discussed (Figure S2).

Antibacterial consumption trends are presented in Table S1. Prior to the intervention in 2022, piperacillin-tazobactam use was increasing while cefazolin and ceftriaxone were declining, suggesting a shift toward broader-spectrum therapy. The intervention was associated with a change in prescribing trends. During the intensive phase, there was an immediate decrease in use of piperacillin-tazobactam (−0.46 DOT/100 OBD; 95%CI −0.86, −0.05; *P* = .027), while there was an increase in ceftriaxone (1.68; 95%CI 0.54, 2.82; *P* = .05) and oral amoxicillin (2.04; 95%CI −0.86, −0.05; *P* = .027). Over time, there was sustained decrease in use of IV flucloxacillin (−0.06; 95%CI −0.10, −0.03; *P* < .001), aminoglycosides (−0.01; 95%CI −0.011, −0.00; *P* < .001), and ampicillin (−0.04; 95%CI −0.06, −0.02; *P* < .001). (Table S1 and Figure S3). When analyzed in aggregate (Table S2 and Figure S4), use of broad-spectrum IV agents remained unchanged, whereas narrow-spectrum IV agents demonstrated a significant reduction (0.16; 95%CI −0.22, −0.09; *P* < .001). Total antibacterials (IV plus oral) following intervention significantly reduced (−1.18 DOT; 95%CI −2.30 to−0.07, *P* = .038, Figure S5).

Mortality did not differ (9.6% intensive vs 10.7% baseline). Median LOS was reduced during the intensive phase (5.5 vs 7.6 days; adj exp coef 0.73; 95%CI 0.59–0.90; *P* = .004), particularly in patients without bacteremia (5.6 [3.4, 10.0] vs 7.9 [4.4, 13.6], adj exp coef 0.75 [0.59, 0.95], *P* = .018). Readmission rates were low (4%–8%) and did not significantly change over the assessed time periods. Readmission with the same infection syndrome was 1% throughout the intervention period. The rate of recommencement of IV therapy was lower during the intensive phase than in the baseline phase (1% vs 7%; 0.18; [0.04, 0.89], *P* = .036) (Table 2).

The intervention generated 4,039 digital communications between the AMS team and general medicine teams in the study period. The estimated pharmacist time commitment was approximately 8–10 hours per week during the intensive phase, and 3–4.5 hours per week during the maintenance phase.

## Discussion

Although our intervention did not further reduce the median IV antimicrobial duration, our ability to further reduce IV duration was likely constrained by strong baseline AMS performance. Our baseline median was already comparable to the postintervention mean IV duration achieved in other face-to-face, general medicine AMS programs.^9^ This aligns with the typical 48–72 hour period required to obtain preliminary microbiology results, evaluate inflammatory markers, and assess clinical stability. Additionally, some IV-to-oral switch opportunities may have been delayed, particularly over weekends when the AMS service was not available, further limiting the potential for earlier transition. As this intervention did not aim to influence prescribing at the point of initiation; optimizing prescribing at this earlier decision point represents an important next step for reducing total treatment duration.

Our pharmacist-led digital model achieved a 78% recommendation acceptance rate, comparable to other in-person handshake stewardship interventions (80%–91%) in general medicine cohorts.^18–20^ Similar acceptance rates (80%) were reported by Neuner et al. with thrice-weekly ID pharmacist and ID physician review,^19^ while higher rates were seen in more intensive daily models (90–91%) described by Morita et al. and Sapozhnikov et al. respectively.^18,21^ While some studies have indicated preference for face-to-face feedback,^18^ our digital model achieved engagement with a more scalable alternative. This model enabled review of a larger number of patients across multiple wards, not only a single patient care unit,^18^ supporting broader stewardship reach.

The intervention was associated with reduced LOS during the intensive phase, particularly among patients without bacteremia. This is a meaningful finding in this patient population, where hospital admissions are often prolonged.^22–24^ This aligns with prior focused AMS initiatives demonstrating shorter LOS in patients with cellulitis,^25^ older adults,^26^ and general hospital inpatients.^27^ We hypothesize that frequent IV-to-oral switch recommendations (89%) increased prescriber confidence for earlier discharge, without increasing readmissions, a conclusion further supported by the reduction in total antibiotic consumption observed in the long-term evaluation. A limitation was the lack of detailed data capturing decision-making contributing to LOS.

Antibacterial utilization trends indicate the intervention reinforced and sustained high-quality prescribing practices. Unlike other studies showing reduction in broad-spectrum antimicrobials,^19,21,28,29^ our site already tightly regulated these agents, with notable decline observed only in piperacillin-tazobactam. Instead, sustained decreases were seen in total antibacterial use following implementation, including narrow-spectrum IV agents (flucloxacillin and ampicillin) without compensatory broad-spectrum increases. This suggests the intervention may have promoted early cessation of therapy.

Implementation of this digital review and communication model enabled consistent, multidisciplinary input while avoiding logistical challenges of daily in-person reviews, which have limited sustainability in other pilots.^18–21,28^ Nonetheless, the model still required substantial time and resources which we recognize may not be practical in other settings. An implementation science-informed approach may help tailor the intervention model to local contexts, identify barriers and facilitators to uptake, and guide adaption of less resource-intensive strategies such as targeting specific infections/antimicrobials or employing less demanding models of audit and feedback to better balance sustainability and workforce capacity.^30^

Limitations of this study include its before-and-after design, which is vulnerable to unmeasured confounding and secular trends. The unchanged median IV duration likely reflects the timing of AMS reviews: recommendations were issued around midday, after the third IV dose had usually been administered, meaning the day was still counted as an IV day even when a switch occurred promptly. This highlights a broader limitation of DOT-based metrics, which are sensitive to dosing times rather than clinical decision-making. Given that total antibacterial use declined after implementation, measures such as total duration of therapy or time to switch may better capture the impact of AMS interventions in future work. There is increasing evidence that shorter therapy durations are as effective as longer durations for common infections seen in general medical units such as community acquired pneumonia.^31^

In summary, this study demonstrates that a pharmacist-led digital AMS model is feasible and associated with high engagement, sustained low use of IV antimicrobials, and reductions in both total antibacterial use and LOS. Future AMS initiatives should consider alternative outcome measures, such as total duration of therapy, to further assess their impact on LOS.

## Supporting information

10.1017/ash.2026.10313.sm001 Khumra et al. supplementary material Khumra et al. supplementary material

## Supplementary material

The supplementary material for this article can be found at https://doi.org/10.1017/ash.2026.10313
