# Supplementary material for: EMERGE: Early antiMicrobial stEwaRdship for GEneral medicine patients—targeting inpatient intravenous therapy greater than 24 hours
Source: Antimicrob Steward Healthc Epidemiol. 2026 Mar 27;6(1):e71. doi: 10.1017/ash.2026.10313 (PMC13104568; doi:10.1017/ash.2026.10313)
Supplement: Khumra et al. supplementary material [file S2732494X26103131sup001.docx]

**SUPPLEMENTARY MATERIALS**

**Figure S1:** Type, frequency and acceptance of AMS recommendations

**
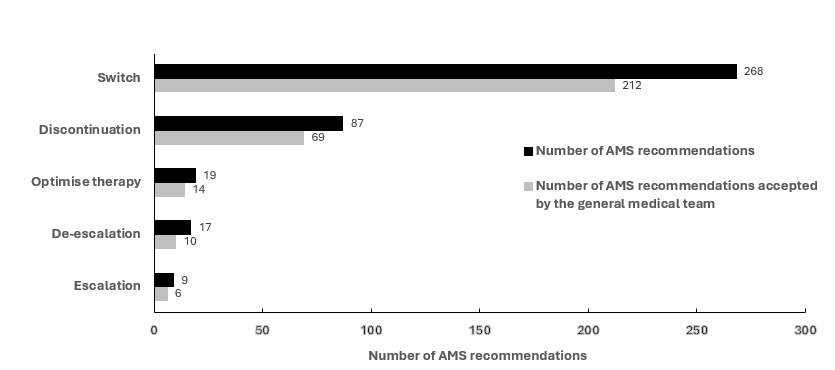
**

**Note.** **Escalation**: broaden the spectrum of activity based on guidelines or microbiology test results; **De-escalation:** narrow the spectrum of activity based on guidelines of microbiology test results; **Discontinuation:** cease an antimicrobial due to unlikely infection, recommended duration of therapy reached, unnecessary spectrum of antimicrobial activity; **Switch:** switch from intravenous to oral antimicrobials, change to alternative intravenous antimicrobial with similar spectrum of activity; **Optimization:** enter cease date in the electronic medical record, modify the dose regimen based on patient or infection-related factors, management of drug-drug interactions, additional microbiology test or therapeutic drug monitoring required.

**Figure S2** Number of AMS recommendation by intravenous antimicrobial

**Figure S3**

**
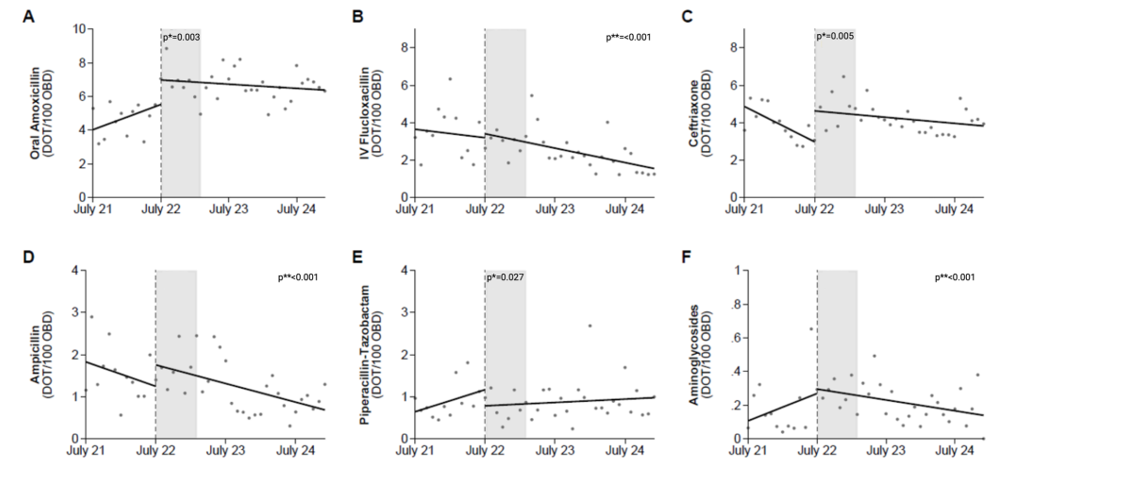
**

**Figure S3**

**
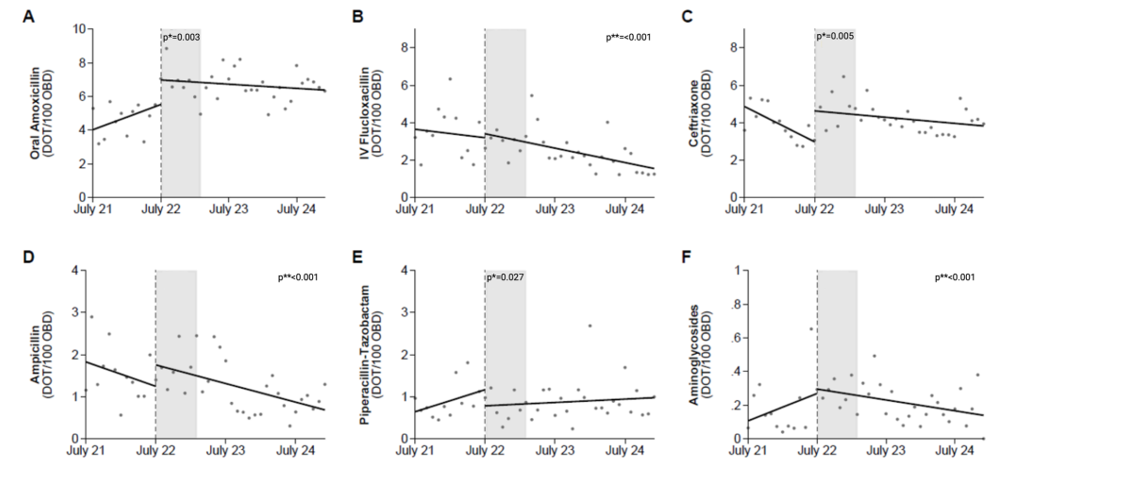
**

**Figure S4** Broad-spectrum vs narrow-spectrum IV antimicrobial consumption dat
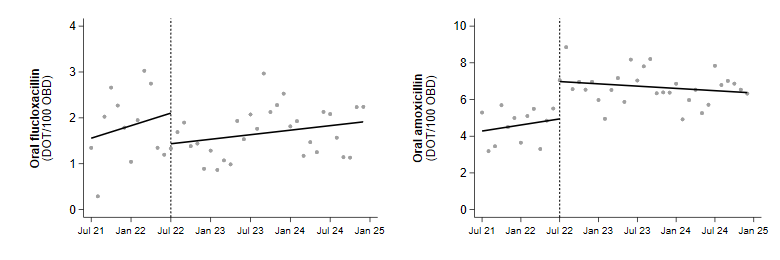
a


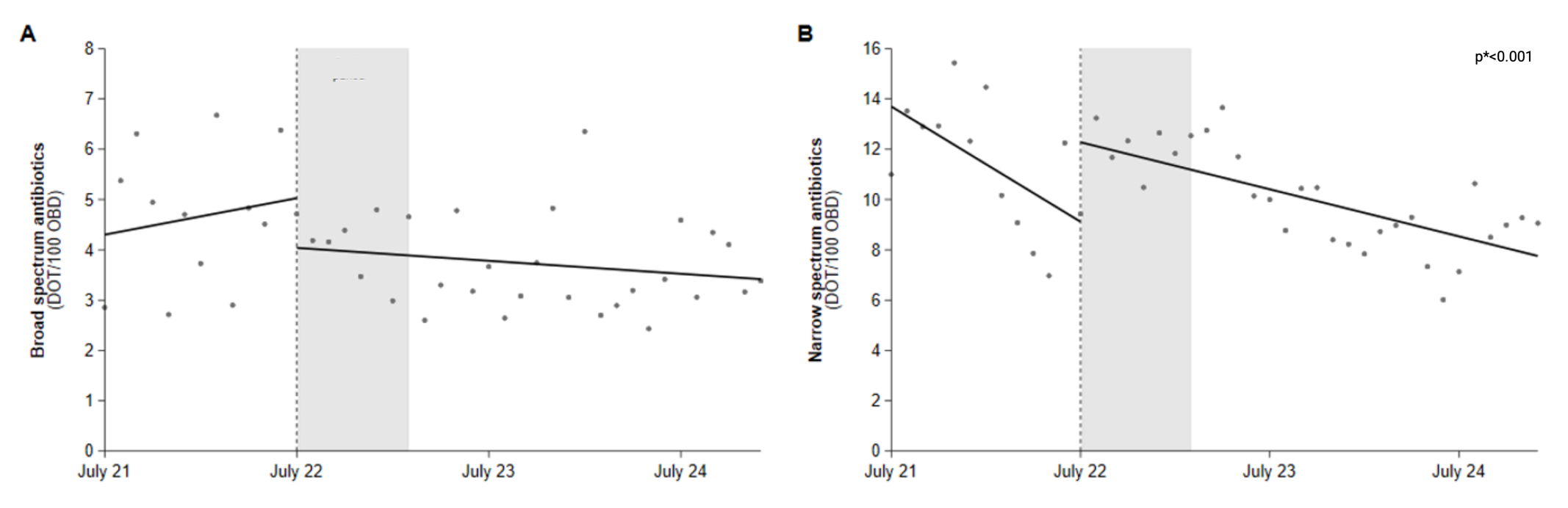


Antibiotic use of IV agents before and after the stewardship intervention. Dotted vertical lines represent start of intervention. The grey section highlights the study period which includes the intensive phase (August–November 2022) and the maintenance phase (November 2022–February 2023). Solid lines represent pre- and post-intervention trends in antibiotic use estimated using interrupted time series analysis. The dots on the graph are raw data points.

*Note: DOT, days of therapy; OBD, occupied bed days;* p* represents statistical significance of change over time in the post-intervention period for narrow-spectrum agents; *Broad-spectrum agents*: amoxicillin/clavulanate, cefepime, ciprofloxacin, piperacillin/tazobactam, vancomycin, meropenem; *Narrow-spectrum agents:* ampicillin, azithromycin, cefazolin, ceftazidime, ceftriaxone, aminoglycoside, flucloxacillin, metronidazole.

**Figure S5** Total antibiotic consumption in the pre and post intervention periods (combined IV and PO)


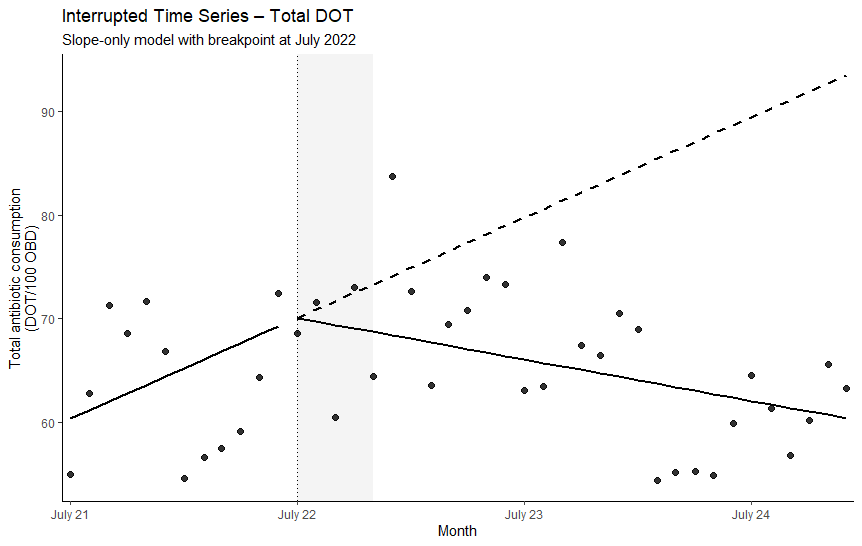


Total antibiotic use (combining IV and PO agents) before and after the stewardship intervention. Dotted vertical lines represent start of intervention. The grey section highlights the study period which includes the intensive phase (August–November 2022) and the maintenance phase (November 2022–February 2023). Solid lines represent pre- and post-intervention trends in antibiotic use estimated using interrupted time series analysis. Dashed lines represent the estimated trajectory of pre-intervention antibiotic usage. The dots on the graph are raw data points.
*Note: DOT, days of therapy; OBD, occupied bed days*

**Table S1** Change over time in antibiotic use for commonly targeted antibiotics, based on DOT/100 OBD/month.

| Antibiotic | Pre intervention – change over time (slope) | | At the time of intervention start (Jul 22) | | Post intervention – change over time | | slope post vs slope pre |
| --- | --- | --- | --- | --- | --- | --- | --- |
|  | Coefficient (95% CI) | p | Coefficient (95% CI) | p | Coefficient (95% CI) | p | p |
| Aminoglycosides | 0.01 (-0.02, 0.04) | 0.371 | 0.04 (-0.21, 0.28) | 0.768 | **-0.01 (-0.01, -0.00)** | **<0.001** | 0.201 |
| Amoxicillin/clavulanate | 0.06 (-0.13, 0.24) | 0.528 | -0.48 (-1.82, 0.86) | 0.469 | -0.01 (-0.03, 0.02) | 0.514 | 0.477 |
| Ampicillin | -0.06 (-0.17, 0.06) | 0.353 | 0.57 (-0.26, 1.40) | 0.174 | **-0.04 (-0.06, -0.02)** | **<0.001** | 0.759 |
| Oral amoxicillin | 0.06 (-0.11, 0.22) | 0.499 | **2.04 (0.74, 3.33)** | **0.003** | -0.02 (-0.06, 0.02) | 0.254 | 0.365 |
| Azithromycin | 0.00 (-0.01, 0.02) | 0.685 | 0.02 (-0.13, 0.17) | 0.810 | -0.00 (-0.01, 0.01) | 0.889 | 0.669 |
| Cefazolin | **-0.16 (-0.23, -0.08)** | **<0.001** | 0.54 (-0.07, 1.14) | 0.079 | -0.01 (-0.02, 0.01) | 0.426 | **<0.001** |
| Cefepime | -0.00 (-0.03, 0.02) | 0.669 | 0.03 (-0.22, 0.28) | 0.806 | 0.00 (-0.01, 0.01) | 0.527 | 0.506 |
| Ceftazidime | -0.01 (-0.03, 0.01) | 0.553 | 0.11 (-0.10, 0.32) | 0.306 | -0.00 (-0.01, 0.01) | 0.717 | 0.733 |
| Ceftriaxone | **-0.16 (-0.28, -0.04)** | **0.010** | **1.68 (0.54, 2.82)** | **0.005** | -0.03 (-0.07, 0.01) | 0.187 | 0.035 |
| Oral/IV Ciprofloxacin | -0.01 (-0.02, 0.00) | 0.135 | 0.07 (-0.02, 0.16) | 0.141 | -0.00 (-0.01, 0.00) | 0.091 | 0.384 |
| IV flucloxacillin | -0.01 (-0.21, 0.19) | 0.899 | 0.00 (-1.57, 1.58) | 0.998 | **-0.06 (-0.10, -0.03)** | **<0.001** | 0.610 |
| Meropenem | 0.01 (-0.03, 0.05) | 0.678 | -0.24 (-0.64, 0.16) | 0.235 | -0.01 (-0.02, 0.00) | 0.205 | 0.465 |
| Metronidazole | -0.02 (-0.07, 0.04) | 0.497 | 0.33 (-0.12, 0.79) | 0.148 | -0.01 (-0.02, 0.00) | 0.059 | 0.825 |
| Piperacillin-tazobactam | **0.05 (0.01, 0.10)** | **0.018** | **-0.46 (-0.86, -0.05)** | **0.027** | 0.01 (-0.01, 0.02) | 0.318 | 0.041 |
| Vancomycin | -0.03 (-0.08, 0.01) | 0.154 | -0.02 (-0.45, 0.41) | 0.918 | -0.01 (-0.03, 0.00) | 0.146 | 0.445 |

*Note. DOT, days of therapy; OBD, occupied bed days****.***

**Table S2** Change over time in use of broad vs narrow-spectrum IV agents, based on DOT/100 OBD/month.

| Antibiotic | Pre intervention – change over time (slope) | | At the time of intervention start (Jul 22) | | Post intervention – change over time | | slope post vs slope pre |
| --- | --- | --- | --- | --- | --- | --- | --- |
|  | Coefficient (95% CI) | P | Coefficient (95% CI) | P | Coefficient (95% CI) | P | p |
| Broad^*^ | 0.07 (-0.15, 0.30) | 0.501 | -1.11 (-2.75, 0.54) | 0.181 | -0.02 (-0.05, 0.01) | 0.141 | 0.391 |
| Narrow^^^ | -0.39 (-0.80, 0.01) | 0.056 | 3.28 (-0.15, 6.71) | 0.060 | **-0.16 (-0.22, -0.09)** | **<0.001** | 0.246 |

Note:

Broad spectrum agents include: amoxicillin/clavulanate, cefepime, ciprofloxacin, piperacillin/tazobactam, vancomycin, meropenem.

Narrow spectrum agents include: ampicillin, azithromycin, cefazolin, ceftazidime, ceftriaxone, aminoglycoside, flucloxacillin, metronidazole.
